# Finite element analysis of the kinematic coupling effect of the joints around talus when Ponseti manipulation

**DOI:** 10.1186/s12891-021-04575-0

**Published:** 2021-08-12

**Authors:** Song-Jian Li, Ben-Chao Shi, Cheng-Long Liu, Yu-Bin Liu

**Affiliations:** 1 Orthopedics Center, Department of Orthopedics and Traumatology, Zhujiang Hospital, Southern Medical University, No.253. Gongye Middle Avenue, Haizhu District, 510280 Guangzhou, Guangdong China; 2 Orthopedics Center, Department of Spinal Surgery, Zhujiang Hospital, Southern Medical University, No.253. Gongye Middle Avenue, Haizhu District, 510280 Guangzhou, Guangdong China

**Keywords:** Kinematic coupling, Tarsal joints, Ponseti manipulation, Finite element analysis

## Abstract

**Background:**

Little information was obtained from the published papers about the kinematic coupling effect between tarsal bones during Ponseti manipulation. The aim was to explore the kinematic coupling effect of the joints around talus, to investigate the kinematic rhythm and coupling relationship of tarsal joints; to clarify the pulling effect on medial ligament of the ankle during the process of Ponseti manipulation.

**Methods:**

The model of foot and ankle was reconstructed from the Chinese digital human girl No.1 (CDH-G1) image database. Finite element analysis was applied to explore the kinematic coupling effect of the joints around talus. The distal tibia and fibula bone and the head of talus were fixed in all six degrees of freedom; outward pressure was added to the first metatarsal head to simulate the Ponseti manipulation. Kinematic coupling of each tarsal joint was investigated using the method of whole model splitting, and medial ligament pulling of the ankle was studied by designing the model of medial ligament deletion during the Ponseti manipulation.

**Results:**

All the tarsal joints produced significant displacement in kinematic coupling effect, and the talus itself produced great displacement in the joint of ankle. Quantitative analysis revealed that the maximum displacement was found in the joints of talonavicular (12.01mm), cuneonavicular (10.50mm), calcaneocuboid (7.97mm), and subtalar(6.99mm).The kinematic coupling rhythm between talus and navicular, talus and calcaneus, calcaneus and cuboid, navicular and cuneiform 1 were 1:12, 1:7, 1:2 and 1:1.6. The results of ligaments pulling showed that the maximum displacement was presented in the ligaments of tibionavicular (mean 27.99mm), talonavicular (21.03mm), and calcaneonavicular (19.18 mm).

**Conclusions:**

All the tarsal joints around talus were involved in the process of Ponseti manipulation, and the strongest kinematic coupling effect was found in the joints of talonavicular, subtalar, calcaneocuboid, and cuneonavicular. The ligaments of tibionavicular, talonavicular, and calcaneonavicular were stretched greatly. It was suggested that the method of Ponseti management was a complex deformity correction processes involved all the tarsal joints. The present study contributed to better understanding the principle of Ponseti manipulation and the pathoanatomy of clubfoot. Also, the importance of cuneonavicular joint should be stressed in clinical practice.

## Background

**Fig. 1:**
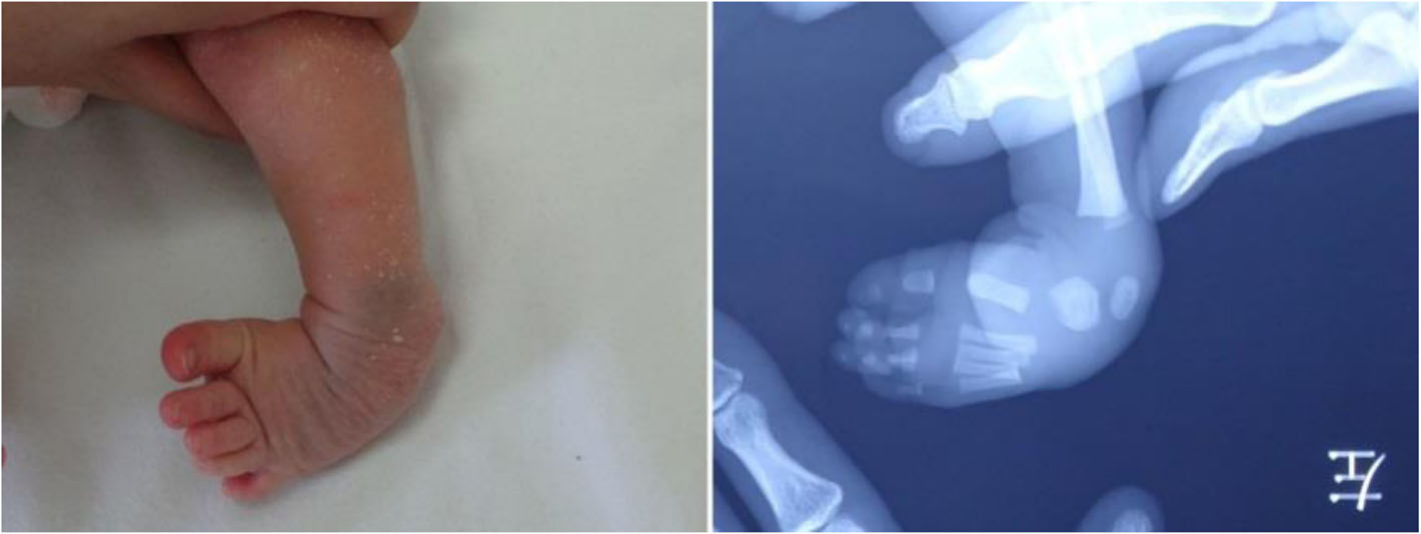
The appearance and abnormal joints were presented for the clubfoot case with 9 days old

Clubfoot is a complex three dimensional foot deformity, and the precise etiology and pathogenesis of this deformity remains unclear [1–4]. Multiple potential risk factors, such as smoking, maternal age, family history, amniocentesis and some selective serotonin reuptake inhibitor exposures, have been reported [5, 6]. The tarsal complex was composed of the subtalar joint (STJ), talonavicular joint (TNJ), and calcaneaocuboid joint (CCJ) [7, 8]. The abnormal arrangement of hindfoot (the anterior portion of calcaneus directly beneath the head of the talus) contributed greatly to the deformities of equinus and varus. The medial displacement of tarsal bones (navicular, cuboid, cuneiforms) was responsible for the deformity of adduction [9]. Published paper about clubfoot cases reported that the cartilaginous structure of the calcaneus was significantly medially rotated (15°) relative to the bimalleolar axis of ankle [10]. Cahuzac et al. [11] reported that an average of 77 ± 19° of the navicular (vs. talus) and an average of 76 ± 19° of the cuboid (vs. calcaneus) were medially displaced on the coronal plane; and an average of 62 ± 27° of the navicular (vs. talus) and 60 ± 12° of the cuboid (vs. calcaneus) were situated downward on the sagittal plane. Guda et al. [12] investigated the morphology and alignment of tarsal bones using 3-dimensional MRI analysis, and the results showed that patients with the medial deviation of talar neck might have the alignment change of navicular bone and distal tibiofibular joint. The above results indicated that the tarsal complex was presented abnormal arrangement in the joints around talus of clubfoot deformity [9, 11–14] (Fig. 1).

**Fig. 2:**
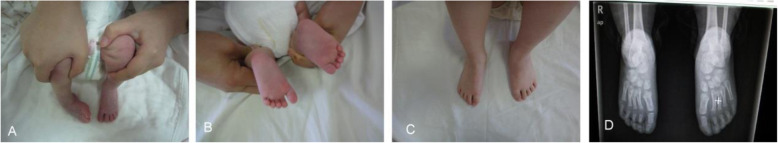
Good result was produced after Ponseti management for clubfoot. Our clubfoot case with 12 days old was treated before (**a**) and after (**b**) Ponseti method. Good morphological and radiological results were presented after 4 years follow-up (**c** and **d**)

Nowadays, Ponseti method has been gradually accepted as the “gold standard” for the initial treatment of clubfoot, and its safety and efficacy has been demonstrated consistently in the past decades [2–4, 9]. Ponseti manipulation of deformity correction was to supinate the forefoot in proper alignment with the hindfoot, and outward pressure was exerted on the first metatarsal to realign simultaneously the talonavicular, the calcaneocuboid, and the subtalar joint [9, 14, 15]. Satisfactory clinical and functional results (Fig. 2) have been demonstrated by long-term follow-up of the patients [3, 12, 13, 16]. The process of Ponseti management was based on the principle of kinematic coupling effect between tarsal bones. Knowledge of the three-dimensional (3D) motion of the tarsal bones was essential for a complete understanding of Ponsei manipulation. Previous researches have measured tarsal joint kinematics based on some in vitro [17, 18] and in vivo [8, 19] techniques, but little information was obtained from the published papers about the kinematic coupling effect between tarsal bones during Ponseti manipulation. The aim of present study was (1) to explore the kinematic coupling effect of the tarsal joints around talus; (2) to investigate the kinematic rhythm and coupling relationship of tarsal joints; (3) to clarify the pulling effect on medial ligament of the ankle during the process of Ponseti manipulation.

## Methods

 The three dimensional model of foot and ankle was reconstructed from the Chinese digital human girl No.1 (CDH-G1) image database. The data base of CDH-G1 was obtained from a baby specimen. It was an image set with slice interval of 1mm and the number of 4265 images. The softwares of Photoshop CS3 (Adobe Company, San Jose, CA, USA), Mimics 17 (Materialise software, Leuven, Belgium), and Geomagic studio 12 were used for the model building, smoothing and optimizing. The processed model was then converted into the software of Hypermesh 13.0 (Altair Company, Troy, MI, USA) and meshed with tetrahedron elements to obtain a solid mesh model. Finally, the solid mesh model was then imported into the software of Abaqus 6.12 (Dassault Systemes Simulia Company, Providence, RI, USA) for material properties setting and ligaments adding. The Young’s modulus of the bone and cartilage were assigned as 38 MPa and 2.3 MPa, while the Poisson’s ratio was 0.3 for bone and 0.4 for cartilage in the established model [20]. The ligaments were modeled as linear springs. A total of 28 ligaments and the Achilles tendon were modeled as linear springs with assigned stiffness values as our previous study reported [15]. The contact behavior between the articulating surfaces was considered as frictionless [21]. In present study, the method of finite element analysis was applied to explore the kinematic coupling effect of the joints around talus during Ponseti manipulation based on the model of foot and ankle from the CDH-G1 image database. For better illustration of kinematic-coupling effect between tarsal bones, the distal tibia and fibula bone and the head of talus were fixed in all six degrees of freedom during the whole test. The outward pressure was added to the first metatarsal head to simulate the Ponseti manipulation. The kinematic coupling of each tarsal joint was investigated using the model splitting method, and the pulling effect of medial ligament was studied by designing the model of medial ligament deletion during the Ponseti manipulation. The pulling effect of the medial ligaments was investigated by measuring the displacement of navicular bone when Ponseti manipulation. The detailed process of the model building and the validation of the established model could be reproduced in the published paper [15]. In present study, the database of CDH-G1 was applied for the establishment of foot and ankle model. This article does not contain any studies with human participants or animals performed by any of the authors. No statement of ethics approval should be declared.

## Results

### Von Mises stress distribution of the tarsal joints around talus

**Fig. 3:**
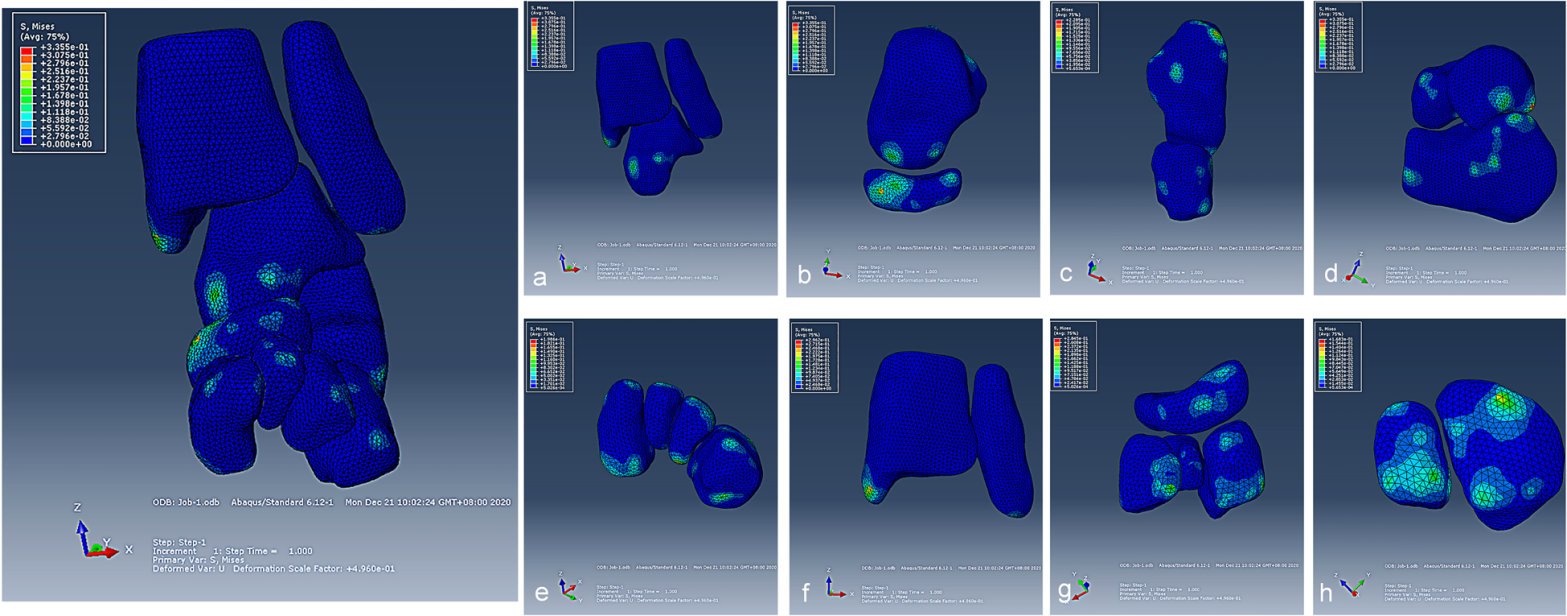
Von Mises stress distribution of the tarsal joints around talus. The Mises stress distribution of the joints of ankle (**a**), talonavicular (**b**), calcaneocuboid (**c**), subtalar (**d**), intercuneiform (**e**), distal tibiofibular syndesmosis (**f**), cuneonavicular (**g**), cuneocuboid (**h**)

The results of established model showed that the stress concentration areas were distributed in medial of the navicular, distal and medial of the tibia, calcaneus, cuneiform and cuboid (Fig. 3). For further analyze the stress distribution of the tarsal joints around talus, we split the whole established model into measurable parts. The finding show that obvious stress concentration areas were presented in talonavicular, calcaneocuboid and subtalar joint, and the stress areas were mainly concentrated on the insertion and original sites of the ligaments (the surface of each tarsal bone). No identified stress concentration was observed in the ankle, distal tibiofibular syndesmosis and intercuneiform joints (Fig. 3 a-h). It was suggested that Ponseti method can correct deformity by pulling ligaments and exerting correction effect by its kinematic coupling relationship.

### The displacement distribution of the tarsal joints around talus

**Fig. 4:**
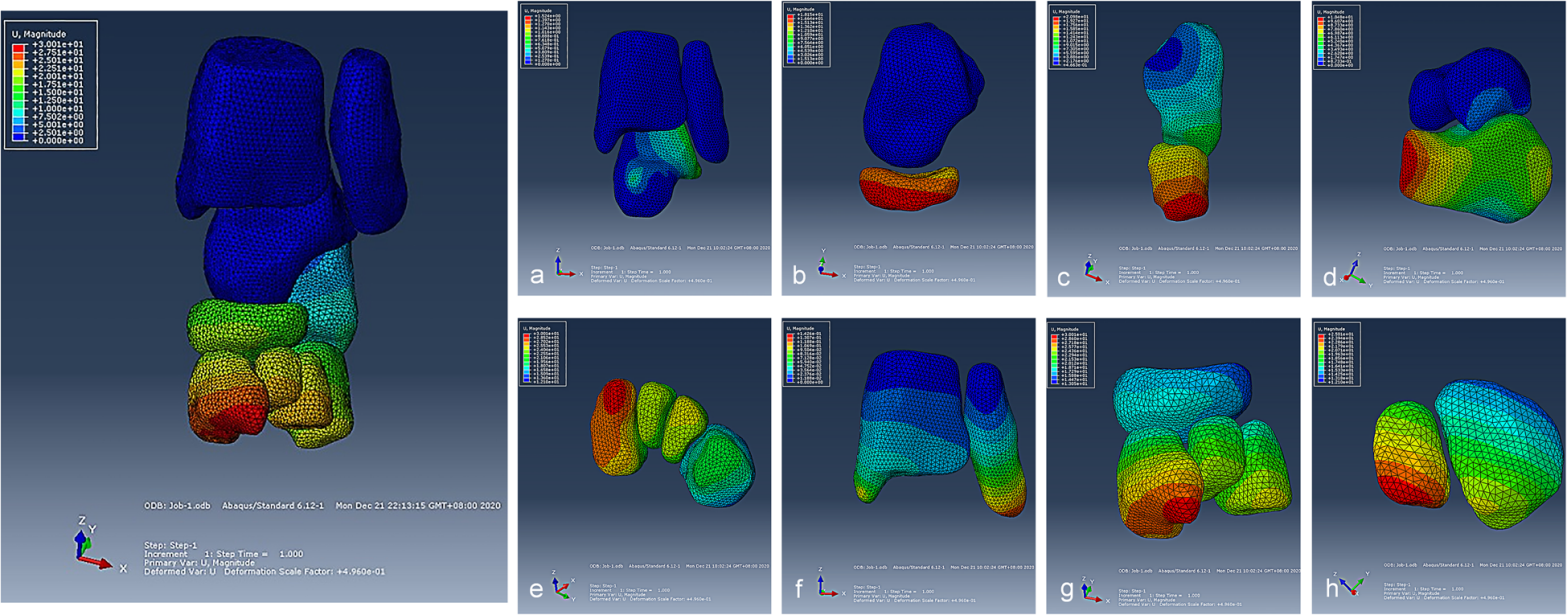
The displacement distribution of the joints around talus. The displacement distribution of the joints of ankle (**a**), talonavicular (**b**), calcaneocuboid (**c**), subtalar (**d**), intercuneiform (**e**), distal tibiofibular syndesmosis (**f**), cuneonavicular (**g**), cuneocuboid (**h**)

**Fig. 5:**
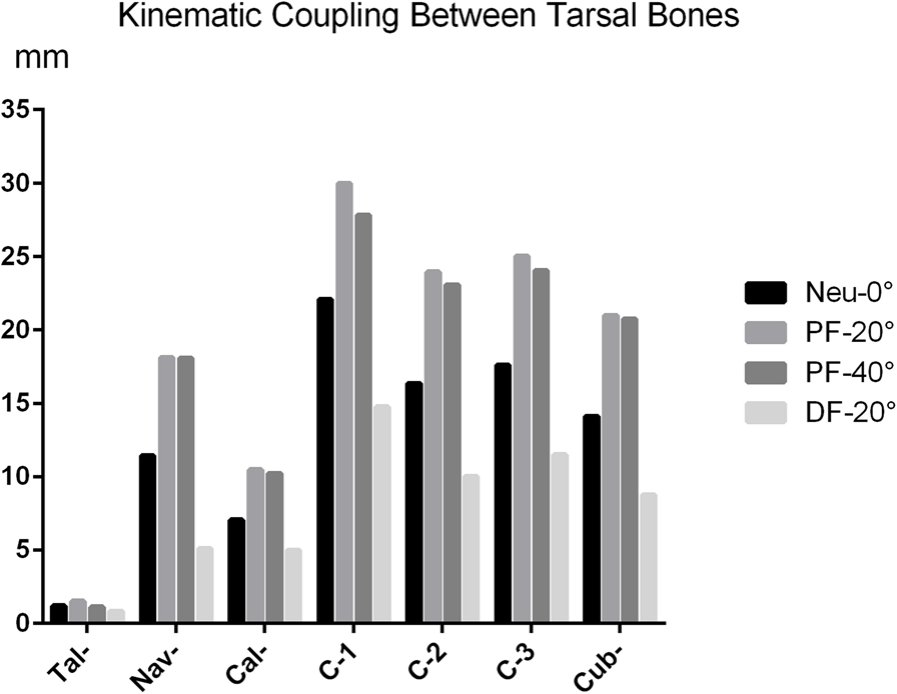
The maximum displacement of tarsal bones at different ankle position including the talus, navicular, calcaneus, 3 cuneiforms and cuboid

The results of displacement of tarsal joints were presented in Fig. 4 under the outward pressure of 3 N. The finding was that the tarsal displacement decrease gradually from distal to proximal, among which the medial cuneiform had the largest displacement and the maximum displacement was distributed on the distal and dorsal side of the medial cuneiform. The calcaneus bone had the least displacement in the whole established model, and the deformation of displacement was mainly concentrated in the front of the calcaneus (Fig. 4). For further explore the relative displacement relationship, we split the whole established model into observable entities to observe the kinematic coupling relationship between tarsal bones. The results of ankle joint showed that the posterior part of talus had a larger displacement than the distal tibiofibula joint during the process of correction (Fig. 4 a). The results of talonavicular joint indicated that the displacement of navicular was larger and showed deeper color cloud image (Fig. 4 b). The results of calcaneocuboid joint showed that the cuboid had larger displacement than calcaneous (Fig. 4 c). The results of subtalar joint showed that the displacement of calcaneus was significantly greater than talus (Fig. 4 d). The results of intercuneiform joints showed that the medial cuneiform (C1) had a largest displacement, and the largest displacement was located in the distal and dorsal side of the medial cuneiform (Fig. 4 e). The results of distal tibiofibular joint showed that minor displacement was found in tibia and fibular, but less than other tarsal bones (Fig. 4 f). The results of cuneonavicular joint showed that navicular and 3 cuneiforms were all involved in the kinematic coupling effect and the maximum displacement was found in medial cuneiform (Fig. 4 g). The results of cuneocuboid joint showed that the lateral cuneiform had larger displacement than cuboid and decreased displacement was found from lateral cuneiform to cuboid in the cuneocuboid joint (Fig. 4 h). For the investigation of the kinematic coupling relationship between tarsal bones, we choose the maximum displacement of each tarsal bone to analyze the kinematic coupling effect. We found that the ankle position of plantar flexion-20 degree (PF-20°) had the maximum displacement including the talus, navicular, calcaneus, 3 cuneiforms and cuboid (Fig. 5).

### Quantitative analysis of kinematic coupling of the tarsal joints around talus

For further analysis the kinematic coupling between tarsal bones, the maximum displacement of each tarsal bone at different ankle position was selected for quantitative analysis. The results showed that the average displacements of talus, navicular, calcaneus, cuneiform-1, cuneiform-2, cuneiform-3 and cuboid were 1.18mm, 13.19 mm, 8.17 mm, 23.67 mm, 18.35 mm, 19.54 mm and 16.14 mm, respectively (Table 1). The average displacement of the talonavicular, cuneonavicular, calcaneocuboid, subtalar, intercuneiform (C1-C2), cuneocuboid and intercuneiform (C2-C3) joints were 12.01mm, 10.50mm, 7.97mm, 6.99mm, 5.32mm, 3.39mm, and 1.19mm, respectively (Table 2). To further investigate the coupling rhythm (odds ratio, OR) between the tarsal bones, we calculated the multiple relationship of maximum displacement with a reference to talus. The finding was that the coupling rhythm between talus and navicular, talus and calcaneus, calcaneus and cuboid, navicular and cuneiform 1, cuneiform 1, 2, and 3, cuneiform and cuboid were 1:12, 1:7, 1:2, 1:1.6, 2:1.6:1.7, and 1.2:1 respectively (Table 1). It was suggested that the joints of talonavicular, subtalar, calcaneocuboid, and cuneonavicular had strong kinematic coupling effect.

**Table 1:** Kinematic coupling between tarsal bones (under the pressure of 3N)

Tarsal bones	Neu-0°		PF-20°		PF-40°		DF-20°		Mean	
	mm	OR	mm	OR	mm	OR	mm	OR	mm	OR
Talus	1.218	1	1.524	1	1.135	1	0.844	1	1.18	1
Navicular	11.42	9.38	18.15	11.91	18.1	15.95	5.092	6.03	13.19	12.29
Calcaneus	7.04	5.78	10.48	6.88	10.21	8.99	4.965	5.88	8.17	7.13
Cuneiform-1	22.07	18.12	30.01	19.70	27.82	24.51	14.78	17.51	23.67	20.5
Cuneiform-2	16.36	13.43	23.95	15.72	23.07	20.33	10.02	11.87	18.35	16.3
Cuneiform-3	17.61	14.46	25.01	16.41	24.04	21.18	11.49	13.61	19.54	17.11
Cuboid	14.12	11.59	20.98	13.77	20.74	18.27	8.738	10.35	16.14	14.35

Cuneiform-1: medial cuneiform; Cuneiform-2: intermedius cuneiform; Cuneiform-3: lateral cuneiform

**Table 2:** Kinematic coupling between different joints (under the pressure of 3N)

Jionts	Neu-0° (mm)	PF-20° (mm)	PF-40° (mm)	DF-20° (mm)	Mean (mm)
Talonavicular	10.20	16.63	16.97	4.25	12.01
Subtalar	5.82	8.96	9.08	4.12	6.99
Calcaneocuboid	7.08	10.50	10.53	3.77	7.97
Cuneonavicular	10.65	11.86	9.72	9.69	10.50
Intercuneiform (1-2)	5.71	6.06	4.75	4.76	5.32
Intercuneiform (2-3)	1.25	1.06	0.97	1.47	1.19
Cuneocuboid	3.49	4.03	3.30	2.75	3.39

Intercuneiform (1-2): Intercuneiform joints between Cuneiform-1 and 2; Intercuneiform (2-3): Intercuneiform joints between Cuneiform-2 and 3

### The pulling effect on medial ligament of the ankle

The pathological anatomy of clubfoot showed that the deltoid, tibionavicular ligament, and the tibialis posterior tendon to be very thick and to merge with the short plantar calcaneonavicular ligament [9, 13]. It was indicated that the ligament contracture contributed greatly to the deformity of clubfoot. We design the models of medial ligaments deletion to explore the pulling effect of Ponseit manipulation on medial ligaments of the ankle. The results showed that the maximum displacement was presented in the models of lack tibionavicular ligament (mean 27.99 mm), lack talonavicular ligament (21.03mm), and lack calcaneonavicular ligament (19.18 mm) (Table 3). It was revealed that the kinematic coupling effect of Ponsei manipulation mainly pulled the ligaments of tibionavicular, talonavicular, and calcaneonavicular.

**Table 3:** The maximum displacement of navicular bone (under the pressure of 3N)

Ligaments	DF-20° (mm)	Neu-0° (mm)	PF-20° (mm)	PF-40° (mm)	Mean (mm)
No Lack of Ligaments	5.09	11.42	18.15	18.10	13.19
Lack of Tibionavicular	23.87	28.52	30.69	28.89	27.99
Lack of Cuneonavicular	6.71	10.29	16.99	16.16	12.54
Lack of anterior tibiotalar	5.14	11.45	18.18	18.13	13.23
Lack of posterior tibiotalar	5.1	11.42	18.18	18.16	13.23
Lack of tibiocalcaneal	5.17	12.46	18.96	18.05	13.66
Lack of calcaneonavicular	7.61	18.75	25.50	24.84	19.18
Lack of talonavicular	13.26	22.44	24.58	23.84	21.03

## Discussion

Published papers reported that good-to-excellent outcome was produced [1, 3, 9, 13], and no limitations in sport performance or activity could be observed for the cases treated using Ponseti method [22]. It was of great importance to illustrate the knowledge of the kinematic coupling effect between tarsal bones, since the diagnosis and the outcome of treatment depended, in part, on the anatomical relationship between these joints. The coupled motion was produced by the interaction between the morphology of the joints, ligament constraints, and total force through the adjacent joints [23]. The main finding of present study was that the joints of talonavicular, subtalar, calcaneocuboid, and cuneonavicular had strong kinematic coupling effect; and the ligaments of tibionavicular, talonavicular, and calcaneonavicular were stretched greatly when Ponseti manipulation.

In present study, the kinematic-coupling effect was defined as the displacement changes of each tarsal bone after outward pressure added. The finding was that the tarsal displacement decreased gradually from distal to proximal, and the calcaneus bone had the minimal displacement in the whole established model. It was suggested that the kinematic coupling of calcaneus was the weakest when Ponseti manipulation. This consisted with the results that residual varus was found in the treated clubfoot and the most important relapses occurred in the hindfoot [2, 24]. That maybe explain why the key manipulation of Posneti method was the adequate abduction of the foot beneath the stabilized talar head [2, 3, 9, 24]. The kinematic coupling of ankle joint showed that the posterior part of talus had a larger displacement than the distal tibiofibular syndesmosis during the process of correction. It was indicated that the position of the talus was not constant and deformation production during the process of deformity correction. Maybe, this explains the flat top talus occurred in 68 and 74 % of the treated cases [16, 25]. The kinematic coupling of tarsal complex showed significantly greater displacement, which indicated that the joints of talonavicular, calcaneocuboid and subtalar were coupled together and showed the important role in the deformity correction. Besides the tarsal complex, the joints of intercuneiform, distal tibiofibular, cuneonavicular and cuneocuboid were all involved in the process of Ponseti manipulation. Roche et al. [26] reported that the surgically treated clubfoot was 25–40 % smaller in the mean talar articular surface area, 78 % smaller in the mean tibiotalar articular surface length difference, and 86 % larger in the mean navicular “flattening index”. A study from Cahuzac et al. [10] reported that the long axis of the osseous nucleus of the talus was medially rotated relative to the cartilaginous anlage (14°), and the cartilaginous structure of the calcaneus medially rotated (15°) relative to the bimalleolar axis. The above results indicated that the deformity correction was a complex procedure with 3 dimensional spatial changes of tarsal bones and the rearrangement between tarsal joints. We found the ankle position of PF-20° had great kinematic coupling effect between tarsal bones. It was indicated that there was kinematic coupling and decoupling effect between tarsal bones when ankle position changed. The locking and unlocking mechanism contributed to the rigidity and flexibility of the foot by changing the direction of the convex curvature axes of the talonavicular and calcaneocuboid articular surface [27, 28]. Published studied reported that six degree-of-freedom coupled motion was identified in the talocrural joint during the dorsiflexion and plantarflexion of the ankle [29, 30].

The pathological anatomy of clubfoot showed that the deltoid, tibionavicular ligament, and the tibialis posterior tendon to be very thick and to merge with the short plantar calcaneonavicular ligament [9, 13]. It was indicated that the ligament contracture of the ankle contributed greatly to the deformity of clubfoot. We chose quantitative analysis of the maximum displacement of each tarsal bone to further analyze the kinematic coupling effect between joints and investigate the pulling effect on the retracting ligaments of the ankle. We found that the joints of talonavicular, subtalar, calcaneocuboid, and cuneonavicular had strong kinematic coupling effect. It was easy to understand that the joints of talonavicular, subtalar and calcaneocuboid were coupled together, and the arrangement had been fully studied in anatomy, kinematic and kinetics [1, 9, 24]. The strongest kinematic coupling effect was found in the joint of talonavicular with the rhythm of 1:12, then the joint of subtalar (1:7). While, the joints of calcaneocuboid and cuneonavicular had the similar kinematic coupling rhythm of 1:2 and 1:1.6 respectively (Table 1). The results of ligaments lack model showed that the tibionavicular, talonavicular, and calcaneonavicular ligaments were mainly pulled with the mean displacement of 27.99mm, 21.03mm and 19.18mm. This was consistent with the findings in the pathoanatomy of clubfoot that the tibionavicular and calcaneonavicular ligaments were very thick and short [14]. We also proved the effectiveness and rationality of Ponseti manipulation on the pulling of contracture ligaments of tibionavicular and calcaneonavicular. Besides, the talonavicular ligament was also stretched greatly with mean displacement of 21.03mm in the model of medial ligament deletion. It was indicated that the pivot around navicular bone (including the joint cuneonavicular and the talonavicular ligament) should be taken great attention in the kinematic coupling effect between tarsal bones.

The merit of present study was that the kinematic coupling effect of the joints around talus during Ponseti manipulation was systematically and innovatively studied in terms of finite element method. This study contributes to better understanding the principle of Ponseti manipulation and the pathoanatomy of clubfoot. The main limitation was that the axial motion of each tarsal bone and the angle changes of tarsal joints were not investigate during the simulated process of deformity correction. Published study reported that the uniaxial motion was shown in talus, and biplanar (sometimes triplanar) translation was exhibited in calcaneus, navicular, and cuboid bones in addition to biaxial rotation [31]. However, it was not the main focus of present study to explore the axial motion of each tarsal bone and the angle changes of tarsal joints when Ponseti manipulation.

## Conclusions

All the tarsal joints around talus (the joints of ankle, talonavicular, calcaneocuboid, subtalar, intercuneiform, distal tibiofibular syndesmosis, cuneonavicular, and cuneocuboid) were involved in the process of Ponseti manipulation, and the strongest kinematic coupling effect was found in the joints of talonavicular, subtalar, calcaneocuboid, and cuneonavicular. The position of the talus was not constant and deformation production when kinematic coupling effect launching, and the ligaments of tibionavicular, talonavicular, and calcaneonavicular were stretched greatly during the process of Ponseti manipulation. It was suggested that Ponseti management was a complex and 3 D deformity correction processes involved all the tarsal bones. The present study contributed to better understanding the principle of Ponseti manipulation and the pathoanatomy of clubfoot. Also, the importance of cuneonavicular joint should be stressed in clinical practice.

## Data Availability

The datasets used and/or analyzed during the current study are available from the corresponding author on reasonable request.

## Abbreviations

STJ: Subtalar joint
TNJ: Talonavicular joint
CCJ: Calcaneaocuboid joint
CDH-G1: Chinese digital human girl No.1
PF: Plantar flexion
3D: Three-dimensional
ROM: Range of motion
C-1: Medial cuneiform
C-2: Intercuneiform
C-3: Lateral cuneiform
